# The study of anthropometric estimates in the visceral fat of healthy individuals

**DOI:** 10.1186/1475-2891-13-46

**Published:** 2014-05-20

**Authors:** Chun-Hao Chen, Yu-Yawn Chen, Chih-Lin Chuang, Li-Ming Chiang, Shu-Min Chiao, Kuen-Chang Hsieh

**Affiliations:** 1 Office of Physical Education, Tunghai University, Taichung City, Taiwan; 2 Department of Physical Education, National Taiwan University of Physical Education and Sport, Taipei City, Taiwan; 3 Department of Radiology, Jen-Ai Hospital, Taichung City, Taiwan; 4 College of Health Science, Movement Activities and Lifetime Fitness Department, East Stroudsburg University of Pennsylvania, East Stroudsburg, PA 18301, USA; 5 Department of Chemical and Materials Engineering, Tunghai University, Taichung City, Taiwan; 6 Office of Physical Education and Sport, National Chung Hsing University, Taichung City, Taiwan; 7 Research Center, Charder Electronic Co., LTD, No. 103, Guozhong Rd., Dail Dist., Taichung City, Taiwan

**Keywords:** Computed tomography, Stepwise linear regression analysis, Subcutaneous fat thickness, Waist circumference, Waist-to-hip ratio

## Abstract

**Background:**

Abdominal visceral fat affects the metabolic processes, and is an important risk factor for morbidity and mortality. The purpose of the study was to develop a quick and accurate estimate in the visceral fat area (VFA) of the L4-L5 vertebrae using anthropometric predictor variables that can be measured conveniently.

**Methods:**

A total of 227 individuals participated in this study and were further divided into a Modeling group (MG) and a Validation group (VG). Anthropometrics measurements (height, weight, waist circumference, hip circumference, age, and subcutaneous fat thickness) and VFA_CT_ were measured using computer assisted tomography for all participants. Multivariate linear regression analysis was applied to the MG to construct a VFA estimator using anthropometric predictor variables and to evaluate its performance using the VG.

**Results:**

The estimate equation obtained from the MG were VFA_ANT_ = -144.66 + 1.84X_1_ + 1.35X_2_ + 0.52X_3_ (r = 0.92, SEE =14.58 cm^2^, *P* < 0.001, *n* = 152). The X_1_, X_2_, and X_3_ variables in the equation were denoted as waist circumference (WC), age, and abdomen subcutaneous fat thickness (AS). In addition, the correlation between VFA_ANT_ and VFA_CT_ showed a high correlation (r = 0.92).

**Conclusion:**

A rapid and accurate VFA estimation can be achieved by using only age, WC, and AS. The approach in the present study provides an easy and reliable estimate that can be applied widely in health and epidemiology studies.

## Introduction

Abdominal fat can be divided into visceral fat and subcutaneous fat. Previous literatures suggest that the abdominal visceral fat affects the metabolic processes and is an important risk factor for morbidity and mortality [1-3]. The abdominal fat can be precisely and reliably measured using magnetic resonance imaging (MRI) or computed tomography (CT) [4,5]. However, the procedure is not only expensive but also limited to hospitals or research centers for clinical and epidemiological studies. To overcome these problems and reduce costs, current research seeks to find more accurate and precise indicators of anthropometric abdominal fat [6-8]. Commonly used anthropometric measures to estimate abdominal visceral fat include body mass index (BMI) [9], waist-to-hip ratio (WHR) [10], waist-to-thigh ratio [10], waist circumference [8,11,12], sagittal abdominal diameter [8,13] and skinfold thickness [14].

Although those anthropometric measures are easy to obtain and are usually highly reproducible, studies that are associated with the research in abdominal visceral fat area (VFA) uses one or more anthropometric measures are inconclusive. Therefore, by using different anthropometric variables to estimate VFA provides different interpretations and physiological significance [13,15]. On the other hand, when too many anthropometric predictor variables are introduced on the right-hand side of a regression model, collinearity becomes an issue. An overly complex model, of which there are more predictor variables to collect, will not necessarily improve the accuracy of estimation [14]. Therefore, it is important to find an optimal set of anthropometric measures that would lead to a high accuracy rate for VFA estimation yet would be easy to obtain and to be reliably interpreted.

In the present study, the CT data on the amount of fat around the human L4-L5 vertebrae were used to estimate VFA (visceral fat area) [1]. Stepwise multivariate linear regression was applied to the modeling group to build a VFA estimate using BMI, height, age, WHR, WC, and subcutaneous fat thickness at several sites as independent predictor variables. The VFA estimation model was then tested using data from the validation group.

## Subjects and methods

### Subjects

A total of 227 Taiwanese adult subjects (Asian) were recruited voluntarily and agreed to participate by signing informed consent forms. A subject pool was generated by posting advertisements about the nature of the study throughout Taiwan. Participants filled out survey information that included demographic information, their physical characteristics and their health status. None of the participants had an endocrine, nutritional or growth disorder and did not have any major chronic diseases. Individuals that had been diagnosed with hypertension, diabetes, cancer, renal dysfunctional liver disease or long-term pulmonary asthma or who were pregnant were excluded from the study. This project was implemented from the Department of Radiology at Taichung Jen-Ai hospital and approved for ethical approval by the Institutional Review Board at the hospital.

### Anthropometry

Participants’ weight was measured using the electronic scale produced by Weight-Tronix (Scale Electronics Development, New York, USA) to the nearest 0.1 kg. Height of the participants was measured in their bare feet using a stadiometer (Holtain, Crosswell, Wales, UK) to the nearest 0.5 cm. Body mass index (BMI) was defined as weight in kilograms divided by height in meters squared. Waist circumference (WC) was measured at the level of the belly button and hip circumference (HC) was measured at the largest circumference point around the hips and butt. Both were measured using a standard tape measure to the nearest 0.1 cm. Each anthropometric measurement was performed by a single well trained observer in the same room. All subjects, wearing a hospital cotton/polyester blend robe with minimal underwear, were measured in private. Waist circumference was measured to the nearest centimeter at the level of the umbilicus with both arms hanging freely at the end of gentle expiration, hip circumference was measured at the spina iliaca anterior superior. By the same observers, the circumferences measurement variability was within 1 mm with tapes calibrated weekly.

### Skinfold measurement

Skinfold thickness was measured using the Lange skinfold caliper (Cambridge Scientifc, Cambridge, MD) at ten different locations on the body, including the abdomen (abdominal skinfold, AS), the suprailiac (suprailiac skinfold, SS) [16], armpit (armpit skinfold, ARS), the triceps (triceps skinfold, TS), the back (back skinfold, BS), the subscapula (subscapula skinfold, SUS), the thigh (thigh skinfold, THS), the chest (chest skinfold, CS), the leg (calf skinfold, CAS) and the chin (chin skinfold, CHS). Each site was measured three times and the average value for each site was used. These measures were taken on the left side of the body early in the morning to standardize the conditions of fluid balance [17].

### Computed tomography

We used a 64-slice CT Scanner (Somatorn Sensation 64 CT system), (Siemens Corp., Germany) together with the operating software (Software Version syngo CT2005A) to scan the abdominal area. Each participant laid down in the center of the CT scanning platform and the lumbar region was scanned. Scanning was performed with voltage of 120Kv, a tube current of 120mAs and an x-ray beam width of 1.5 mm; scanning time was 0.5 s. Additionally, slice thickness was 5 mm, and images were obtained at 2 mm intervals. The image reconstruction kernel index was B20. The image was furthered processed using the commercial software 3D-Doctor Ver. 3.5 (Able software Corp., USA). Procedures and specifications recommended by previous literature were used to scan images at the navel height and to color the areas of abdominal visceral fat and abdominal subcutaneous fat followed by an estimate of the fat areas of the coloring areas (i.e. visceral fat area, hereafter denoted as VFA_CT_) [5]. The threshold range for adipose tissue in CT was approximately (-260 ± 3) to (-10 ± 3) Hu. Before the analysis, CT scans were performed to L4-L5 abdominal visceral fat area two times with a three day interval on five participants to determine the reliability of the measurements.

### Experiment procedure

The experiment began 2:00 pm every afternoon during June 2008 to May 2011. The measuring sequences were body weight, height, waist circumference, hip circumference, skinfold thickness, and CT scan. The CT, anthropometric, and skinfold measurement were all performed by the same radiologist and research assistant.

### Statistical analysis

The demographic statistics of visceral fat area (VFA) were measured by CT (VFA_CT_) are presented using mean ± SD for age, anthropometric measures (i.e., height, weight, BMI, waist and hip circumferences) and skinfold measurement in the modeling and validation groups by CT measurements. The minimum and maximum values were reported in parentheses.

Multivariate stepwise linear regression was used for age, sex, and other anthropometric predictor variables (height, weight, BMI, WC, HC, WHR, AS, SS, ARS, TS, BS, SS, THS, CS, CAS, CHS) and VFA_CT_ was used as the response variable. Stepwise regression analysis with forward (F_in_ = 4.00) and backward (F_out_ =3.99) independent variable selection was used for all of the anthropometric variables in the modeling group (MG) to obtain the estimated variables. The regression coefficient, standard estimate error (SEE), coefficient of determination was further used to construct the VFA estimate equation (VFA_ANT_). When independent variables are closely related to one another, we may consider removing the variable with variance inflation factor (VIF) ≥ 3 from the estimate equation. Bland-Altman Plot [18] was performed to evaluate the goodness-of-fit between the results of VFA_ANT_ estimate equation and VFA_CT_ in the modeling and validation group (VG). Further, pre- and post-test CT data from five patients were used to confirm the test-retest reliability of the measurements. Statistical significance was set at *P* < 0.05 for all tests. All analyses were carried out using SPSS for WINDOWS (Version 16.0; SPSS Inc, Chicago).

## Results

The subjects were randomly divided into the MG (2/3 total subjects) and VG (1/3 total subjects). The modeling group consist 87 males (age: 28.56 ± 11.20 years, BMI: 24.68 ± 4.04 kg/m^2^) and 65 females (age: 33.28 ± 16.23 years, BMI: 23.15 ± 3.93 kg/m^2^); the validation group consisted of 43 males (age: 26.13 ± 12.63 years, BMI: 25.27 ± 3.02 kg/m^2^) and 32 females (age: 27.07 ± 8.11 years, BMI: 24.00 ± 5.60 kg/m^2^). The results of the VFA_CT_ in male (51.49 ± 40.71 cm^2^) and female (58.80 ± 37.88 cm^2^) participants were normally distributed. The VFA_CT_ and other circumference measurement results are indicated in Table 1. The correlation coefficient and Cronbach’s alpha of the CT scans measured from the five participants’ VFA_CT_ were 0.99 and 0.99 respectively.

**Table 1:** **Physical characteristics of the study participants**^**1**^

	**Modeling group (*n*** **= 152)**				
**Variable**	**Male**	**(*n*** **= 87)**	**Female**	**(*n*** **= 65)**	***P***^***2***^
Age (year)	28.56 ± 11.20	(*18.7, 65.5)*	*33.*28 ± 16.23	(18.5, 74.8)	**
Height (cm)	173.39 ± 7.85	(165.0, 195.5)	160.84 ± 6.24	(163.6,174.0)	***
Weight (kg)	74.41 ± 14.17	(50.0, 121.6)	59.66 ± 9.35	(52.0, 98.0)	***
BMI (kg/m^2^)	24.68 ± 4.04	(16.3, 39.9)	23.15 ± 3.93	(16.2, 37.8)	*
Waist circumference (cm)	82.41 ± 10.82	(69.0, 122.5)	81.11 ± 10.39	(68.0, 115.0)	NS
Hip circumference (cm)	98.50 ± 7.98	(87.5, 120.0)	97.81 ± 7.86	(87.5, 125.5)	NS
ACSA_CT_ (cm)	478.61 ± 142.62	(284.4,1004.8)	448.48 ± 122.94	(288.7,875.2)	NS
VFA_CT_ (cm)	51.49 ± 40.71	(6.5, 179.5)	58.80 ± 37.88	(13.0, 218.8)	NS
SFA_CT_ (cm)	112.56 ± 97.66	(2.7, 513.2)	165.59 ± 99.25	(20.5, 488.8)	***
Waist-hip ratio (WHR)	0.83 ± 0.06	(0.72, 1.02)	0.83 ± 0.06	(0.73, 1.08)	NS
Subcutaneous fat thickness (SFT)					
Abdominal, AS (mm)	18.34 ± 13.29	(2.5, 59.5)	23.91 ± 14.12	(1.0, 62.0)	**
Suprailiac, SS (mm)	18.56 ± 13.33	(1.8, 59.8)	24.02 ± 14.11	(1.0, 62.5)	**
Armpit, ARS (mm)	15.67 ± 11.48	(4.0, 59.0)	23.88 ± 14.37	(3.0, 55.0)	***
Triceps, TS (mm)	16.08 ± 11.77	(4.0, 58.0)	24.07 ± 14.51	(3.0, 56.0)	***
Subscapular, SUS (mm)	15.99 ± 11.53	(4.0, 60.0)	23.72 ± 14.58	(3.0, 58.0)	***
Back, BS (mm)	15.91 ± 11.56	(4.0, 59.0)	24.02 ± 14.53	(3.0, 56.3)	***
Thigh, THS (mm)	12.41 ± 8.63	(3.0, 38.0)	19.63 ± 14.30	(3.0, 77.0)	***
Calf, CAS (mm)	12.65 ± 8.84	(3.0, 42.0)	19.07 ± 12.92	(3.5, 55.0)	***
Chin, CHS (mm)	12.69 ± 8.77	(2.5, 40.5)	19.02 ± 12.97	(3.0, 56.0)	***
Chest, CS (mm)	12.58 ± 8.72	(2.8, 40.2)	19.25 ± 12.72	(3.2, 55.0)	***
	**Validation group (*n*** **= 75)**				
	**Male**	**(*n*** **= 43)**	**Female**	**(*n*** **= 32)**	***P***^***2***^
Age (year)	26.13 ± 12.63	(18.2, 70.8)	27.07 ± 8.11	(18.9, 46.4)	NS
Height (cm)	174.64 ± 8.51	(159.4, 197.0)	161.35 ± 5.53	(156.0,173.5)	***
Weight (kg)	77.03 ± 10.19	(61.5, 102.0)	62.58 ± 15.52	(47.6, 106.0)	***
BMI (kg/m^2^)	25.27 ± 3.02	(20.3, 32.6)	24.00 ± 5.60	(17.3, 38.0)	NS
Waist circumference (cm)	84.82 ± 10.00	(68.0, 109.0)	82.36 ± 13.00	(67.6, 122.0)	NS
Hip circumference (cm)	100.13 ± 5.68	(88.0, 113.6)	99.64 ± 11.64	(86.0, 129.0)	NS
ACSA_CT_ (cm)	485.40 ± 119.58	(323.7, 795.6)	472.40 ± 164.72	(294.7,995.2)	NS
VFA_CT_ (cm)	53.09 ± 40.84	(8.2, 166.2)	58.98 ± 36.17	(18.7, 171.1)	NS
SFA_CT_ (cm)	123.26 ± 90.85	(10.5, 388.0)	171.34 ± 118.66	(8.1, 506.8)	NS
Waist-hip ratio (WHR)	0.85 ± 0.06	(0.73, 1.00)	0.82 ± 0.06	(0.73, 0.96)	NS
Subcutaneous fat thickness (SFT)					
Abdominal, AS (mm)	18.33 ± 14.64	(3.0, 57.0)	26.02 ± 14.82	(3.0, 60.0)	*
Suprailiac, SS (mm)	18.53 ± 14.56	(3.0, 56.5)	26.42 ± 14.76	(3.0, 55.0)	*
Armpit, ARS (mm)	17.90 ± 14.27	(3.5, 61.0)	24.58 ± 13.92	(3.0, 55.0)	*
Triceps, TS (mm)	18.27 ± 14.24	(4.0, 60.5)	24.39 ± 13.72	(2.0, 55.0)	NS
Subscapular, SUS (mm)	18.24 ± 14.12	(3.0, 62.0)	24.47 ± 13.81	(4.0, 57.0)	NS
Back, BS (mm)	18.14 ± 14.19	(3.5, 61.2)	24.48 ± 13.77	(3.0, 55.7)	*
Thigh, THS (mm)	12.80 ± 9.37	(2.5, 42.0)	20.11 ± 11.23	(2.0, 52.5)	**
Calf, CAS (mm)	13.08 ± 9.54	(3.0, 42.2)	20.41 ± 12.54	(2.5, 52.0)	**
Chin, CHS (mm)	12.89 ± 9.17	(3.0, 41.0)	20.09 ± 12.43	(3.0, 53.0)	**
Chest, CS (mm)	12.92 ± 9.34	(2.8, 41.8)	20.20 ± 12.38	(2.5, 52.5)	**

^1^All values are $\overset{¯}{x}\;\pm \;\mathrm{SD}$; minimum and maximum in parentheses;

^2^Significantly different from male (one-factor ANOVA): NS, not statistically significant;

*P < 0.05; **P < 0.01; ***P < 0.001;

BMI: Body mass index; SFA, subcutaneous fat area; VFA, visceral fat area; ACSA, abdominal cross-sectional area.

The Correlation coefficient between VFA_CT_ and predictor variables were as follows: Age (r = 0.70), Height (r = -0.13), Weight (r = 0.44), BMI (r = 0.63), WC (r = 0.75), HC (r = 0.54), WHR(r = 0.74), AS (r = 0.80), SS (r = 0.72), ARS (r = 0.81), TS (r = 0.57), SUS (r = 0.76), BS (r = 0.76), THS (r = 0.49), CAS (r = 0.45), CHS (r = 0.66), CS (r = 0.74), Sex (r = -0.06). ARS and Sex are the highest and lowest predictor variables in VFA_CT_ (Table 2).

**Table 2:** **Correlation coefficient matrix between VFA**_**CT **_**and predictor variables in the modeling group (*n *** **= 152)**

	**VFA**_**CT**_	**ARS**^**1**^	**AS**^**1**^	**SUS**^**1**^	**BS**^**1**^	**WC**	**CS**^**1**^	**WHR**	**SS**^**1**^	**Age**	**CHS**^**1**^	**BMI**	**TS**^**1**^	**HC**	**THS**^**1**^	**CAS**^**1**^	**Wt**	**Sex**
**ARS**^**1**^	.81^**^																	
**AS**^**1**^	.80^**^	.86^**^																
**SUS**^**1**^	.76^**^	.82^**^	.78^**^															
**BS**^**1**^	.76^**^	.83^**^	.80^**^	.92^**^														
**WC**	.75^**^	.63^**^	.56^**^	.57^**^	.58^**^													
**CS**^**1**^	.74^**^	.84^**^	.80^**^	.80^**^	.74^**^	.44^**^												
**WHR**	.74^**^	.64^**^	.58^**^	.59^**^	.55^**^	.80^**^	.54^**^											
**SS**^**1**^	.72^**^	.76^**^	.83^**^	.73^**^	.74^**^	.48^**^	.74^**^	.48^**^										
**Age**	.70^**^	.60^**^	.67^**^	.62^**^	.56^**^	.28^**^	.79^**^	.42^**^	.68^**^									
**CHS**^**1**^	.66^**^	.76^**^	.74^**^	.73^**^	.73^**^	.48^**^	.72^**^	.50^**^	.72^**^	.54^**^								
**BMI**	.63^**^	.57^**^	.46^**^	.50^**^	.54^**^	.87^**^	.34^**^	.56^**^	.37^**^	.16^**^	.43^**^							
**TS**^**1**^	.57^**^	.68^**^	.69^**^	.66^**^	.59^**^	.31^**^	.74^**^	.37^**^	.56^**^	.37^**^	.16	.43^**^						
**HC**	.54^**^	.44^**^	.38^**^	.38^**^	.44^**^	.87^**^	.22^**^	.39^**^	.34^**^	.09	.31^**^	.86^**^	.16^*^					
**THS**^**1**^	.49^**^	.59^**^	.65^**^	.61^**^	.56^**^	.25^**^	.69^**^	.34^**^	.69^**^	.64^**^	.66^**^	.11	.77^**^	.10				
**CAS**^**1**^	.45^**^	.54^**^	.61^**^	.55^**^	.50^**^	.20^*^	.67^**^	.28^**^	.65^**^	.62^**^	.61^**^	.10	.74^**^	.07	.85^**^			
**Wt**	.44^**^	.38^**^	.22^**^	.35^**^	.35^**^	.77^**^	.15	.44^**^	.12	-.02	.18^*^	.84^**^	.09	.81^**^	-.10	-.08		
**Sex**	-.06	-.06	-.23^**^	.01	-.07	.06	-.07	.10	-.31^**^	-.14	-.26^**^	.15	-.11	.01	-37^**^	-.22^**^	.48^**^	
**Ht**	-.13	-.18^*^	-.30^**^	-.13	-.18^*^	.12	-.24^**^	-.05	-.35^**^	-.27^**^	-.34^**^	.06	-.23^**^	.23^**^	-.38^**^	-.33^**^	.58^**^	.66^**^

^1^subcutaneous fat thickness; AS: abdominal skinfold, SS: suprailiac skinfold, ARS: armpit skinfold, TS: triceps skinfold, BS: back skinfold, SUS: subscapula skinfold, THS: thigh skinfold, CS: chest skinfold, CAS: calf skinfold, CHS: chin skinfold; WC: waist circumference; HC: hip circumference; WHR: waist-to-hip ration; Sex(0: female, 1: male); Ht, height; Wt, weight; BMI, body mass index; *, **.Correlation is significant at the 0.05, 0.01 level (2-tailed).

Age, height, weight, BMI, Sex, WC, HC, WHR, and subcutaneous fat thickness (all sites) were selected as predictor variables and VFA as dependent variable for multivariate stepwise linear regression analysis. Age, WC, and AS were the three predictor variables that composed the best model for predicting the VFA. The estimate equation is shown in Eq. (1).

(1) $$\begin{array}{l} {\mathrm{VFA}}_{\mathrm{ANT}}=-144.66+1.84\;X_{1}+1.35\;X_{2}+0.52\;X_{3}\\ \left(r=0.92,\;\mathrm{SEE}=14.58\;{\mathrm{cm}}^{2},\;P<0.001,\;n=152\right) \end{array}$$

The variance inflation factor (VIF) of WC, AS, and Age as predictors for VFA_ANT_ were 1.6, 2.6, 1.9 respectively. All VIF values for WC, Age, and AS were less than 3 indicating that there was no bias due to collinearity. Assuming a medium effect size, 3 predictor variables, 90 observations (100 preferred) were needed to reach 80% power at a 0.05 significance level [19]. After applying Eq. (1) to residual analysis (not shown in text), no significant trends were reported. Other than the three predictor variables, we added another three variables (BMI, WHR, BS) acquired through regression analysis by gradually adding the variables into linear regression analysis. After performing the analysis six times, a better understanding of the collinearity and coefficient of determination between variables were observed in Table 3. When BMI, WHR or BS were added as additional predictor variables in the present model, there was a slight increase in the correlation coefficient for VFA_ANT_ and a slight decrease in the SEE. VIF increased from 2.6 to 8.4 which demonstrated high collinearity. As result, the predictor variables that demonstrated collinearity are not to be included into the estimate equation.

**Table 3:** **Multiple regression analysis results for waist circumference (WC) measured with anthropometric measures as predictor variable and VFA**_**CT **_**as response variable (Modeling group)**

**Cumulative dependent variables used in model (*n*** **= 152)**								
**Waist**	**Age**	**AS**^**1**^	**BMI**	**WHR**	**BS**^**1**^	**Intercept**	**SEE (cm**^**2**^**)**	**r**^**2**^
2.72 ± 0.21(1.0)^**^	-	-	-	-	-	-167.84 ± 17.33^**^	27.296	0.528
2.14 ± 0.13(1.1)^**^	1.64 ± 0.10(1.1)^**^	-	-	-	-	-165.98 ± 10.36^**^	16.322	0.833
1.84 ± 0.15(1.6)^**^	1.35 ± 0.13(1.9)^**^	0.52 ± 0.15(2.6)^**^	-	-	-	-144.66 ± 11.72^**^	15.741	0.844
1.61 ± 0.27(5.1)^**^	1.38 ± 0.13(1.9)^**^	0.52 ± 0.15(2.6)_**_	0.67 ± 0.67(4.4)	-	-	-142.54 ± 11.91^**^	15.741	0.846
1.12 ± 0.35(8.4)^*^	1.33 ± 0.13(2.0)^**^	0.47 ± 0.15(2.6)^*^	1.10 ± 0.68(4.8)	88.75 ± 37.89(3.4)^*^	-	-182.27 ± 20.96^**^	15.517	0.852
1.12 ± 0.35(8.4)^*^	1.33 ± 0.13(2.0)^**^	0.41 ± 0.23(6.5)	1.08 ± 0.69(4.8)	88.30 ± 38.03(3.4)^*^	0.08 ± 0.23(5.5)	-181.51 ± 21.14^**^	15.565	0.852

Regression coefficient estimate ± SEE (VIF, variance inflation factor); r^2^, determination coefficient; *P < 0.05; **P < 0.001; ^1^ subcutaneous fat thickness; AS: abdominal skinfold, BS: back skinfold.

**Figure 1:**
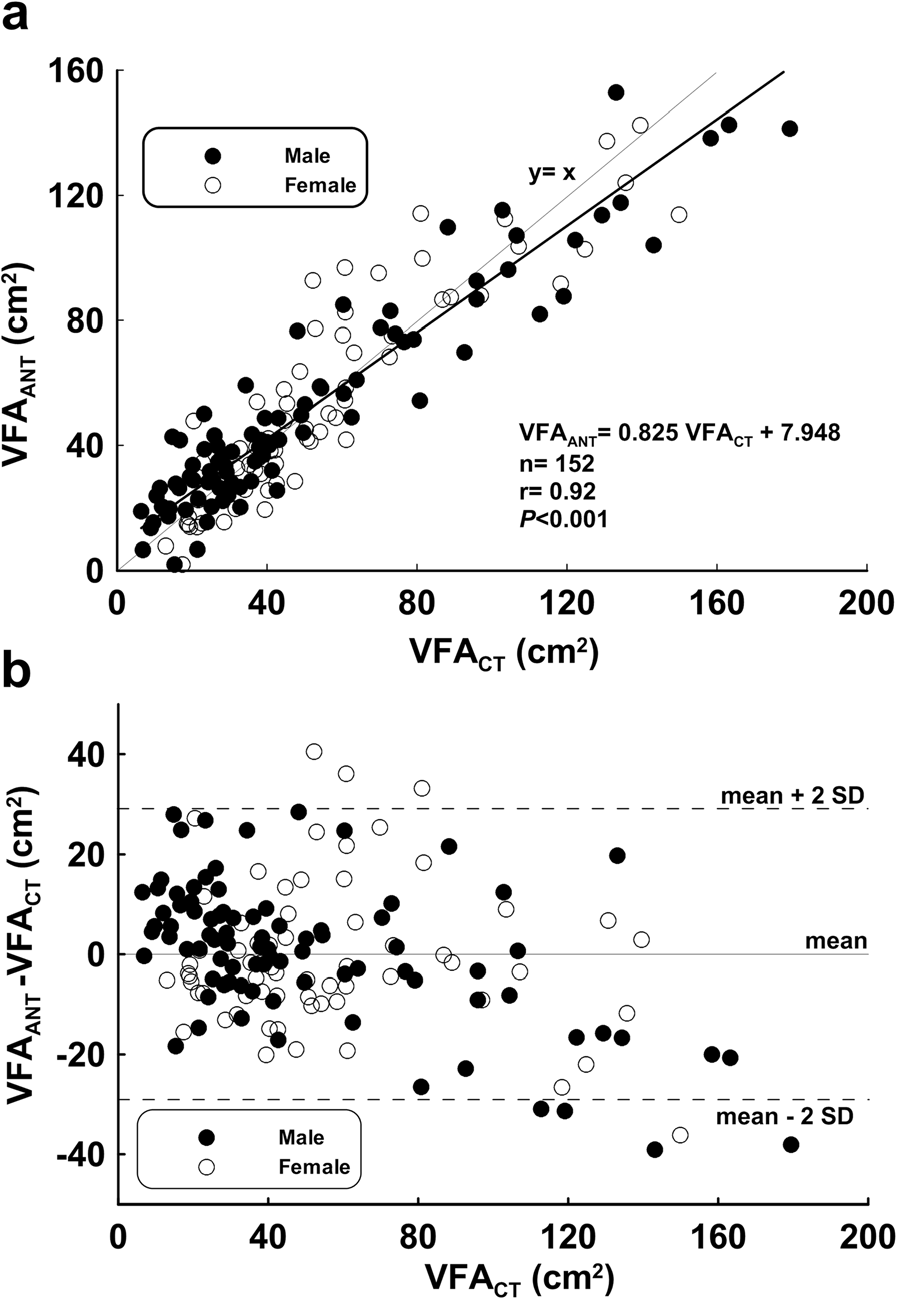
**Modeling group (a) Scatter chart and regression line (b) Bland-Altman Plot (mean: 0.0 cm**^**2**^**, mean – 2 SD: -28.80 cm**^**2**^**, mean + 2 SD: 28.80 cm**^**2**^**).**

Figure 1a shows a scatter plot of VFA_ANT_ estimates and VFA_CT_ values of the modeling group. The regression line is VFA_ANT_ = 0.83 VFA_CT_ + 7.95, r = 0.92, *P* < 0.001. Figure 1b shows the agreement in VFA between VFA_ANT_ and VFA_CT_ by using the Bland and Altman analysis in modeling group. The mean differences in VFA between the VFA_ANT_ and VFA_CT_ methods were ± 28.80 cm^2^.

**Figure 2:**
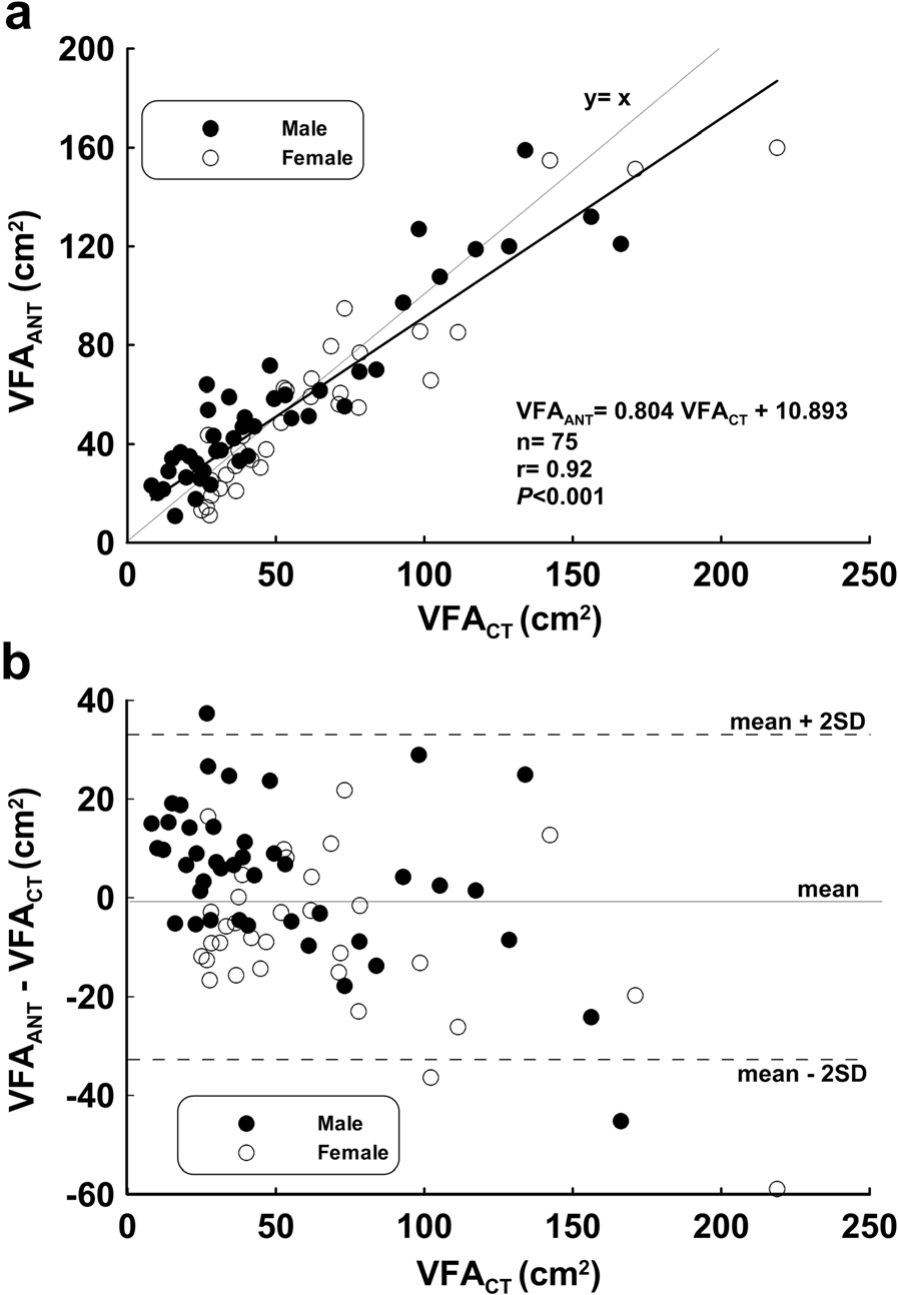
**Validation group (a) Scatter chart and regression line (b) Bland-Altman Plot (mean: -0.37 cm**^**2**^**, mean – 2 SD: -33.44 cm**^**2**^**, mean + 2 SD: 32.70 cm**^**2**^**).**

We applied Eq. (1) to compute the VFA_ANT_ for the validation group, and compared the estimates with VFA_CT_ values (Figure 2b). The regression line was VFA_ANT_ = 0.80 VFA_CT_ + 10.89, r = 0.92, *P* < 0.001. Figure 2b represented the agreement in VFA between VFA_ANT_ and VFA_CT_ and was compared by using the Bland and Altman Plot analysis in the validation group. The mean differences in VFA between the VFA_ANT_ and VFA_CT_ methods were -0.37 ± 33.06 cm^2^, respectively.

## Discussion

In the present study, the modeling group was sufficient (*n* = 152) for the proposed analysis. VFA_ANT_ estimator (r > 0.92, *P* < 0.001) also fared well when compared with prior studies (e.g., Naqaiet *et al*. [20] (r = 0.90-0.92), Demura *et al*. [21] (r = 0.87- 0.90), Ryo *et al*. [22] (r = 0.88)). Our estimator also had a lower SEE (14.58 cm^2^) when compared with Brundavani *et al*. [15] (38.7 and 29.0 cm^2^ for males and females respectively). The results of Bland & Altman plot from the VFA estimate equation in MG did not show any systematic error. In the VG, the standard deviation (-0.37 cm^2^) was near zero with data sets not normally distributed, which indicates neither systematic nor proportional error of the model was observed.

Several factors affect how a regression model performs [23]: (a) cross-validation, (b) a model is deemed unreliable if its VIF is greater than 10, (c) the sample size, (d) the choice of independent predictor variables, (e) and the prediction error or standard error of estimate (SEE). Anthropometric measures were used because they were easy to collect and also biologically relevant as independent predictor variables for estimating VFA_ANT_.

Previous studies focus on VFA estimation used anthropometric measures such as WC, HC, WHR, subcutaneous fat (AS, CS), age, and BMI separately or collectively. While VFA estimation was easy and fast by using a single predictor variable, this lead to a higher estimation error. On the other hand, VFA estimation is inconvenient when the model used too many predictor variables, or relied on expensive technologies. For example, the VFA estimator developed by Demura and Sato [21] used total torso fat that can only be obtained using Dual-energy X-ray absorptiometry (DXA) or Bioelectrical impedance analysis (BIA). Furthermore, total torso fat measurement using BIA was difficult [24]; its large measurement error in turn also affects the accuracy of VFA estimation. Estimating torso fat weight using BIA was a difficult task [24] so there was even larger error when used in VFA estimation. Demura *et al.*[21] also proposed using sagittal diameter, WC, subcutaneous fat thickness (CS, AS, SUS) and left leg and torso fat fractions as predictor variables for VFA estimation. Similar to the present model, their approach also used WS and AS. Nonetheless, left leg and torso fat fractions were difficult to measure [24]. Their model also had a higher number of predictor variables. These issues seriously limit the practical applications of the VFA estimation model.

Adding these additional predictor variables made the model more complex and there was only modest gain in the accuracy of VFA_ANT_ estimation. Thus, we recommend using only WC, Age, and AS for VFA_ANT_ estimation. Bonora *et al.*[14] also pointed out that when more than three predictor variables were used for VFA estimation, there was no obvious decrease in SEE. For some scenarios, it is possible to increase SEE. The adjusted r^2^ decreased gradually or approached a fixed constant. We observed the same effect in the present study.

Among the anthropometric predictor variables examined in the study, many predictor variables (ARS, AS, SUS, BS, WC, CS, WHR, SS, Age) have high correlation (r > 0.7) with VFA_CT_. Other studies have confirmed an existing high positive correlation between VFA_CT_ and WC and WHR. These predictor variables have been widely used in VFA estimation studies. Due to the high correlation between WC and WHR, the two predictor variables are rarely used in the same estimation model, lest collinearity affects the regression estimate.

Age was used for VFA estimation in Seidell *et al*. [10], Naqai *et al*. [20], Demura and Sato [21]. These studies determined correlations between VFA and age for male and female participants were 0.54 and 0.62 respectively, which are similar to the data presented in the study. These three predictor variables can also be obtained easily. In summary, VFA estimation based on Age, WC, and AS is highly accurate, reproducible, and practical.

Several published studies on VFA estimate equations also consider sex as one of the predictor variables [15,21]. However, in the present study, the correlation between sex and VFA_CT_ showed a weak correlation between participants (r = -0.06). Based on the results of the correlation coefficient matrix, weight was the only highly correlated independent variable when compared with sex (r = 0.48). The study further confirmed the effect of sex in VFA estimate by dividing male and female participants ’independent variables and acquiring male and female VFA estimate equation (not shown in text) via regression analysis. When comparing male and female data sets (together and apart), the predictor variables, determination coefficient, regression coefficient, and SEE values were fairly close and no significant differences were found. Hence, sex was omitted from VFA estimate.

There was a high positive correlation (r > 0.7, *P* < 0.01) between VFA_CT_ and subcutaneous fat thickness at the AS, ARS, SUS, BS, CS and SS sites. These six predictor variables were also highly correlated with each other (r > 0.7, *P* < 0.01). Thus, multivariate stepwise regression analysis was chosen to retain AS and exclude the other five predictor variables.

The study has several limitations. First, the participants in the study were young (mean age: 29.2 ± 12.9 years) and the proportion of VFA >100 cm^2^ was small (14.5%), the present study may have difficulty adapting the regression equation for middle-aged people who have a higher proportion of VFA >100 cm^2^ than young people. Second, the data are limited to the Taiwanese population which may have different VFA characteristics than other populations. Third, the number of participants was another limiting factor to the study. During the recruitment process, we interviewed each participant by making sure they are all well and healthy before recruiting them into the study, thus only allowing these participants may be the cause of the decreased sample size. Fourth, the subjects in the study were well and healthy individuals and cannot be applied to the clinical environment. The main focus of the study is to apply the simplified VFA measuring method to healthy individuals and contribute to the field of preventive medicine. In the future, extending the research to more participants and different age groups may enhance the understanding of the research.

## Conclusions

This study demonstrated that rapid and accurate VFA estimation can be achieved using only Age, WC, and AS. Our approach provides an easy and reliable estimate that can be applied widely in health and epidemiology. Using too many anthropometric predictor variables will not increase the precision for VFA estimation.

## Abbreviations

ACSA: Abdominal cross-sectional area; ARS: Armpit skinfold; AS: Abdominal skinfold; BIA: Bioelectrical impedance analysis; BMI: Body mass index; BS: Back skinfold; CAS: Calf skinfold; CHS: Chin skinfold; CS: Chest skinfold; CT: Computed tomography; DXA: Dual-energy X-ray absorptiometry; MG: Modeling group; MRI: Magnetic Resonance; SFA: Subcutaneous fat area; SFT: Subcutaneous fat thickness; SS: Suprailiac skinfold; SUS: Subscapula skinfold; THS: Thigh skinfold; TS: Triceps skinfold; VFA: Visceral fat area; VIF: Variance inflation factor; VG: Validation group; WC: Waist circumference; WHR: Waist-to-hip ratio.

## Competing interest

All authors declare no competing financial interests in relation to the work described.

## Authors’ contributions

CHC and YYC carried out the anthropometry studies, participated in the sequence alignment and drafted the manuscript. CLC, LMC and SMC carried out the operation of instruments. KCH conceived of the study, and participated in its design and coordination and helped to draft the manuscript and performed the statistical analysis. All authors read and approved the final manuscript.
